# Tetra-μ-oxido-tetra­kis{dioxido[3-(2-pyrid­yl)-1*H*-pyrazole]molybdenum(VI)}

**DOI:** 10.1107/S1600536809031717

**Published:** 2009-08-15

**Authors:** Dacheng Li, Ying Liu, Peihai Wei, Bo Hu, Xiutang Zhang

**Affiliations:** a College of Chemistry and Chemical Engineering, Liaocheng University, Liaocheng 252059, People’s Republic of China; b Advanced Material Institute of Research, Department of Chemistry and Chemical Engineering, ShanDong Institute of Education, Jinan 250013, People’s Republic of China

## Abstract

In the title compound, [Mo_4_O_12_(C_8_H_7_N_3_)_4_], the Mo^VI^ ion has a distorted octa­hedral coordination completed by two terminal O atoms, two μ-oxide atoms and two N atoms from one 3-(2-pyrid­yl)-1*H*-pyrazole ligand. It is noteworthy that in the tetranuclear unit (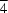 symmetry), any three Mo^VI^ atoms define a plane, and the fourth lies 1.8 (1) Å out of that plane. The degree of linearity of the oxide bridges between two Mo atoms is 175.38 (13)°. Moreover, the N—H group forms an intra­molecular hydrogen bond (four per mol­ecule).

## Related literature

For the properties and potential medical applications of polyoxometalate clusters, see: Pope & Müller (1991 ▶); Khenkin & Neumann (2008 ▶); Zhang *et al.* (2006 ▶, 2007 ▶, 2009 ▶). For Mo—O and Mo—N distances, see: Rana *et al.* (2003 ▶). For general background, see: Mezei *et al.* (2007 ▶).
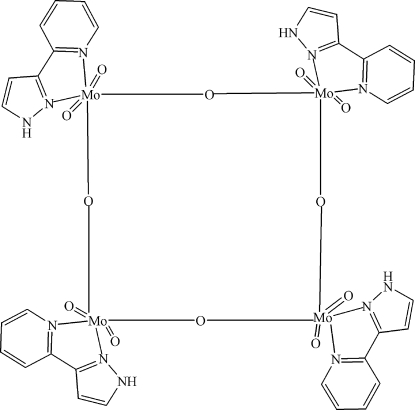


## Experimental

### 

#### Crystal data

[Mo_4_O_12_(C_8_H_7_N_3_)_4_]
*M* *_r_* = 1156.42
Tetragonal, 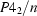

*a* = 14.4412 (16) Å
*c* = 9.094 (2) Å
*V* = 1896.6 (5) Å^3^
*Z* = 2
Mo *K*α radiation
μ = 1.37 mm^−1^
*T* = 298 K
0.12 × 0.10 × 0.08 mm

#### Data collection

Bruker APEXII CCD area-detector diffractometer
Absorption correction: multi-scan (*SADABS*; Bruker, 2001 ▶) *T*_min_ = 0.853, *T*_max_ = 0.898
7579 measured reflections
1675 independent reflections
1316 reflections with *I* > 2σ(*I*)
*R*_int_ = 0.036

#### Refinement

*R*[*F* ^2^ > 2σ(*F* ^2^)] = 0.024
*wR*(*F* ^2^) = 0.064
*S* = 1.00
1675 reflections
139 parameters
1 restraint
H atoms treated by a mixture of independent and constrained refinement
Δρ_max_ = 0.35 e Å^−3^
Δρ_min_ = −0.35 e Å^−3^

### 

Data collection: *APEX2* (Bruker, 2004 ▶); cell refinement: *SAINT-Plus* (Bruker, 2001 ▶); data reduction: *SAINT-Plus*; program(s) used to solve structure: *SHELXS97* (Sheldrick, 2008 ▶); program(s) used to refine structure: *SHELXL97* (Sheldrick, 2008 ▶); molecular graphics: *SHELXTL* (Sheldrick, 2008 ▶); software used to prepare material for publication: *SHELXTL*.

## Supplementary Material

Crystal structure: contains datablocks global, I. DOI: 10.1107/S1600536809031717/zq2001sup1.cif

Structure factors: contains datablocks I. DOI: 10.1107/S1600536809031717/zq2001Isup2.hkl

Additional supplementary materials:  crystallographic information; 3D view; checkCIF report

## Figures and Tables

**Table 1:** Hydrogen-bond geometry (Å, °)

*D*—H⋯*A*	*D*—H	H⋯*A*	*D*⋯*A*	*D*—H⋯*A*
N2—H1*A*⋯O2^i^	0.94 (5)	1.86 (5)	2.783 (4)	168 (4)

Symmetry code: (i) 
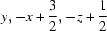
.

**Fig. 1.**
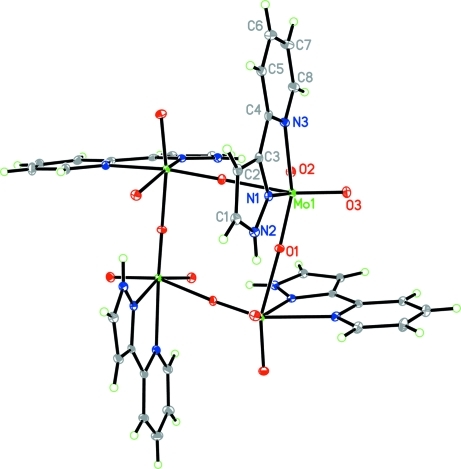
A view of the title compound with the unique atom-labelling scheme. Displacement ellipsoids are drawn at the 30% probability level.

**Fig. 2.**
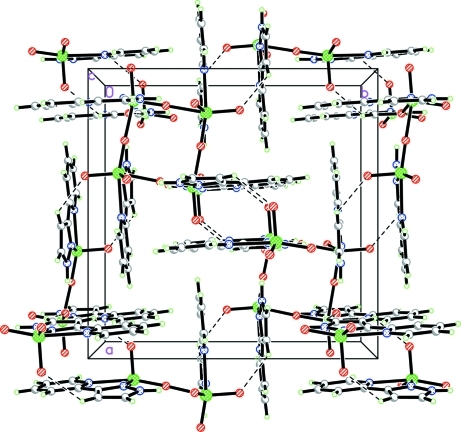
The packing diagram of the title compound with the hydrogen bonds of N2—H1A···O2.

## supplementary crystallographic information

### Comment

The design and synthesis of polyoxometalate clusters has attracted continuous research interest not only because of their appealing structural and topological novelty, but also due to their unusual optical, electronic, magnetic, and catalytic properties, as well as their potential medical application (Pope et al.; Khenkin et al.; Zhang et al. (2007); Zhang et al. (2006); Zhang et al. (2009). In the present paper, we describe the synthesis and structural characterization of tetrakis((µ-oxo)-bis(3-(2-pyridyl)pyrazole)molybdenum(vi)).

In the asymmetric unit of complex I, there exhibit one 3-(2-pyridyl)pyrazole ligand and one molybdenum oxide Mo^VI^O_3_, Fig. 1. The Mo^VI^ ion surrounded by one 3-(2-pyridyl)pyrazole ligand is hexa-coordinated by four oxygen atoms and two nitrogen atoms, with distorted octahedral coordination sphere. The bond distances of Mo—O and Mo—N are in the normal range compared to the reported complexes containing the N—Mo—O atoms (Rana et al.). It is worthy noting that the simple basic Mo_3_HL units are assembled to form one 8-MC-4 complex, which could be described as `folded' with two adjacent Mo_3_ planes forming a dihedral angle of about 38.65°. Moreover, the N—H group forms a very nice intramolecular hydrogen bond (4 per molecule), shown in Fig. 2.

### Experimental

A mixture of 3-(2-pyridyl)pyrazole (1 mmoL) and molybdenum trioxide (1 mmoL) in 10 ml distilled water sealed in a 25 ml Teflon-lined stainless steel autoclave was kept at 433 K for three days. Colourless crystals suitable for an X-ray experiment were obtained. Anal. Calc. for C_32_H_28_Mo_4_N_12_O_12_: C 33.22, H 1.90, N 14.53%; Found: C 33.13, H 1.79, N 14.32%.

### Refinement

All hydrogen atoms bound to carbon were refined using a riding model with C—H = 0.93 Å and *U*_iso_ = 1.2*U*_e__q_ (C) for aromatic atoms. The H atom on nitrogen was located from difference density maps and was refined with a distance restraint of N–H = 0.97 (1) Å.

### Crystal data

**Table:**

[Mo_4_O_12_(C_8_H_7_N_3_)_4_]	*D*_x_ = 2.025 Mg m^−^^3^
*M**_r_* = 1156.42	Mo *K*α radiation, λ = 0.71073 Å
Tetragonal, *P*4_2_/*n*	Cell parameters from 1675 reflections
Hall symbol: -P 4bc	θ = 2.0–25.0°
*a* = 14.4412 (16) Å	µ = 1.37 mm^−^^1^
*c* = 9.094 (2) Å	*T* = 298 K
*V* = 1896.6 (5) Å^3^	Block, colourless
*Z* = 2	0.12 × 0.10 × 0.08 mm
*F*(000) = 1136	

### Data collection

**Table:**

Bruker APEXII CCD area-detector diffractometer	1675 independent reflections
Radiation source: fine-focus sealed tube	1316 reflections with *I* > 2σ(*I*)
graphite	*R*_int_ = 0.036
φ and ω scans	θ_max_ = 25.0°, θ_min_ = 2.0°
Absorption correction: multi-scan (*SADABS*; Bruker, 2001)	*h* = −17→16
*T*_min_ = 0.853, *T*_max_ = 0.898	*k* = −12→17
7579 measured reflections	*l* = −9→10

### Refinement

**Table:**

Refinement on *F*^2^	Primary atom site location: structure-invariant direct methods
Least-squares matrix: full	Secondary atom site location: difference Fourier map
*R*[*F*^2^ > 2σ(*F*^2^)] = 0.024	Hydrogen site location: inferred from neighbouring sites
*wR*(*F*^2^) = 0.064	H atoms treated by a mixture of independent and constrained refinement
*S* = 1.00	*w* = 1/[σ^2^(*F*_o_^2^) + (0.0295*P*)^2^ + 2.0306*P*] where *P* = (*F*_o_^2^ + 2*F*_c_^2^)/3
1675 reflections	(Δ/σ)_max_ = 0.001
139 parameters	Δρ_max_ = 0.35 e Å^−^^3^
1 restraint	Δρ_min_ = −0.35 e Å^−^^3^

### Special details

**Table:**

Geometry. All e.s.d.'s (except the e.s.d. in the dihedral angle between two l.s. planes) are estimated using the full covariance matrix. The cell e.s.d.'s are taken into account individually in the estimation of e.s.d.'s in distances, angles and torsion angles; correlations between e.s.d.'s in cell parameters are only used when they are defined by crystal symmetry. An approximate (isotropic) treatment of cell e.s.d.'s is used for estimating e.s.d.'s involving l.s. planes.
Refinement. Refinement of *F*^2^ against ALL reflections. The weighted *R*-factor *wR* and goodness of fit *S* are based on *F*^2^, conventional *R*-factors *R* are based on *F*, with *F* set to zero for negative *F*^2^. The threshold expression of *F*^2^ > σ(*F*^2^) is used only for calculating *R*-factors(gt) *etc*. and is not relevant to the choice of reflections for refinement. *R*-factors based on *F*^2^ are statistically about twice as large as those based on *F*, and *R*- factors based on ALL data will be even larger.

### Fractional atomic coordinates and isotropic or equivalent isotropic displacement parameters (Å^2^)

**Table:**

	*x*	*y*	*z*	*U*_iso_*/*U*_eq_	
Mo1	0.642839 (18)	0.904662 (19)	0.19761 (3)	0.02233 (12)	
N1	0.61994 (19)	0.90081 (18)	0.4404 (3)	0.0257 (6)	
N2	0.6811 (2)	0.8938 (2)	0.5516 (3)	0.0311 (7)	
N3	0.4862 (2)	0.8976 (2)	0.2532 (3)	0.0314 (7)	
O1	0.76035 (15)	0.88536 (16)	0.2362 (2)	0.0302 (5)	
O2	0.61894 (17)	0.86807 (17)	0.0193 (3)	0.0359 (6)	
O3	0.63381 (18)	1.02324 (17)	0.1907 (3)	0.0370 (6)	
C1	0.6375 (3)	0.8821 (3)	0.6815 (4)	0.0345 (9)	
H1	0.6656	0.8765	0.7731	0.041*	
C2	0.5439 (3)	0.8800 (2)	0.6536 (4)	0.0354 (9)	
H2A	0.4963	0.8726	0.7215	0.042*	
C3	0.5355 (2)	0.8917 (2)	0.4995 (4)	0.0283 (8)	
C4	0.4595 (2)	0.8907 (2)	0.3953 (4)	0.0321 (8)	
C5	0.3667 (3)	0.8828 (2)	0.4346 (5)	0.0413 (10)	
H5	0.3492	0.8796	0.5329	0.050*	
C6	0.3014 (3)	0.8798 (3)	0.3248 (5)	0.0519 (11)	
H6	0.2391	0.8724	0.3477	0.062*	
C7	0.3291 (3)	0.8878 (3)	0.1802 (5)	0.0504 (11)	
H7	0.2853	0.8870	0.1054	0.060*	
C8	0.4208 (3)	0.8969 (3)	0.1471 (5)	0.0423 (10)	
H8	0.4386	0.9027	0.0492	0.051*	
H1A	0.746 (3)	0.894 (3)	0.540 (5)	0.080*	

### Atomic displacement parameters (Å^2^)

**Table:**

	*U*^11^	*U*^22^	*U*^33^	*U*^12^	*U*^13^	*U*^23^
Mo1	0.02453 (18)	0.02550 (18)	0.01698 (17)	0.00112 (12)	0.00115 (11)	0.00047 (12)
N1	0.0347 (15)	0.0237 (14)	0.0187 (14)	0.0010 (12)	−0.0011 (12)	−0.0010 (11)
N2	0.0367 (17)	0.0338 (16)	0.0227 (16)	0.0002 (14)	0.0014 (13)	−0.0026 (13)
N3	0.0298 (16)	0.0315 (16)	0.0328 (16)	0.0037 (13)	−0.0038 (13)	−0.0050 (13)
O1	0.0245 (12)	0.0387 (14)	0.0273 (13)	0.0000 (11)	0.0026 (10)	0.0028 (11)
O2	0.0466 (15)	0.0403 (14)	0.0209 (13)	−0.0016 (12)	−0.0022 (11)	0.0006 (11)
O3	0.0448 (15)	0.0294 (13)	0.0368 (15)	0.0040 (11)	0.0047 (12)	0.0037 (11)
C1	0.044 (2)	0.039 (2)	0.0201 (19)	0.0018 (17)	0.0019 (16)	0.0017 (16)
C2	0.041 (2)	0.034 (2)	0.031 (2)	0.0022 (17)	0.0135 (17)	0.0045 (16)
C3	0.0357 (19)	0.0203 (17)	0.0289 (19)	0.0051 (14)	0.0086 (16)	−0.0006 (14)
C4	0.036 (2)	0.0216 (17)	0.039 (2)	0.0017 (15)	0.0111 (17)	0.0006 (16)
C5	0.034 (2)	0.035 (2)	0.055 (3)	0.0049 (17)	0.0090 (19)	0.0055 (19)
C6	0.033 (2)	0.055 (3)	0.067 (3)	0.0006 (19)	0.002 (2)	0.003 (2)
C7	0.033 (2)	0.067 (3)	0.051 (3)	0.005 (2)	−0.010 (2)	−0.007 (2)
C8	0.036 (2)	0.046 (2)	0.044 (2)	0.0043 (18)	−0.0021 (18)	−0.0070 (19)

### Geometric parameters (Å, °)

**Table:**

Mo1—O3	1.719 (2)	C1—C2	1.375 (5)
Mo1—O2	1.740 (2)	C1—H1	0.9300
Mo1—O1	1.755 (2)	C2—C3	1.416 (5)
Mo1—O1^i^	2.207 (2)	C2—H2A	0.9300
Mo1—N1	2.233 (3)	C3—C4	1.449 (5)
Mo1—N3	2.320 (3)	C4—C5	1.392 (5)
N1—C3	1.340 (4)	C5—C6	1.374 (6)
N1—N2	1.346 (4)	C5—H5	0.9300
N2—C1	1.349 (4)	C6—C7	1.379 (6)
N2—H1A	0.94 (5)	C6—H6	0.9300
N3—C8	1.350 (5)	C7—C8	1.364 (6)
N3—C4	1.353 (5)	C7—H7	0.9300
O1—Mo1^ii^	2.207 (2)	C8—H8	0.9300
			
O3—Mo1—O2	104.69 (12)	N2—C1—C2	107.4 (3)
O3—Mo1—O1	103.82 (11)	N2—C1—H1	126.3
O2—Mo1—O1	109.26 (11)	C2—C1—H1	126.3
O3—Mo1—O1^i^	159.53 (10)	C1—C2—C3	105.4 (3)
O2—Mo1—O1^i^	86.03 (10)	C1—C2—H2A	127.3
O1—Mo1—O1^i^	88.51 (13)	C3—C2—H2A	127.3
O3—Mo1—N1	92.85 (10)	N1—C3—C2	109.3 (3)
O2—Mo1—N1	152.17 (11)	N1—C3—C4	115.3 (3)
O1—Mo1—N1	86.65 (10)	C2—C3—C4	135.3 (3)
O1^i^—Mo1—N1	71.30 (9)	N3—C4—C5	121.7 (4)
O3—Mo1—N3	88.74 (11)	N3—C4—C3	114.1 (3)
O2—Mo1—N3	89.78 (11)	C5—C4—C3	124.2 (3)
O1—Mo1—N3	153.15 (10)	C6—C5—C4	118.4 (4)
O1^i^—Mo1—N3	73.68 (9)	C6—C5—H5	120.8
N1—Mo1—N3	68.85 (10)	C4—C5—H5	120.8
C3—N1—N2	106.7 (3)	C5—C6—C7	119.5 (4)
C3—N1—Mo1	122.3 (2)	C5—C6—H6	120.3
N2—N1—Mo1	130.4 (2)	C7—C6—H6	120.3
N1—N2—C1	111.2 (3)	C8—C7—C6	120.0 (4)
N1—N2—H1A	124 (3)	C8—C7—H7	120.0
C1—N2—H1A	124 (3)	C6—C7—H7	120.0
C8—N3—C4	118.9 (3)	N3—C8—C7	121.4 (4)
C8—N3—Mo1	121.8 (3)	N3—C8—H8	119.3
C4—N3—Mo1	119.3 (2)	C7—C8—H8	119.3
Mo1—O1—Mo1^ii^	175.38 (13)		

Symmetry codes: (i) −*y*+3/2, *x*, −*z*+1/2; (ii) *y*, −*x*+3/2, −*z*+1/2.

### Hydrogen-bond geometry (Å, °)

**Table:**

*D*—H···*A*	*D*—H	H···*A*	*D*···*A*	*D*—H···*A*
N2—H1A···O2^ii^	0.94 (5)	1.86 (5)	2.783 (4)	168 (4)

Symmetry codes: (ii) *y*, −*x*+3/2, −*z*+1/2.
